# Regeneration of myelin sheaths of normal length and thickness in the zebrafish CNS correlates with growth of axons in caliber

**DOI:** 10.1371/journal.pone.0178058

**Published:** 2017-05-25

**Authors:** Marja J. Karttunen, Tim Czopka, Marieke Goedhart, Jason J. Early, David A. Lyons

**Affiliations:** 1 Centre for Neuroregeneration, MS Society Centre for Translational Research, Euan MacDonald Centre for Motor Neurone Disease Research, University of Edinburgh, Edinburgh, United Kingdom; 2 Institute of Neuronal Cell Biology, Technische Universität München, München, Germany; Instituto Cajal-CSIC, SPAIN

## Abstract

Demyelination is observed in numerous diseases of the central nervous system, including multiple sclerosis (MS). However, the endogenous regenerative process of remyelination can replace myelin lost in disease, and in various animal models. Unfortunately, the process of remyelination often fails, particularly with ageing. Even when remyelination occurs, it is characterised by the regeneration of myelin sheaths that are abnormally thin and short. This imperfect remyelination is likely to have implications for the restoration of normal circuit function and possibly the optimal metabolic support of axons. Here we describe a larval zebrafish model of demyelination and remyelination. We employ a drug-inducible cell ablation system with which we can consistently ablate 2/3rds of oligodendrocytes in the larval zebrafish spinal cord. This leads to a concomitant demyelination of 2/3rds of axons in the spinal cord, and an innate immune response over the same time period. We find restoration of the normal number of oligodendrocytes and robust remyelination approximately two weeks after induction of cell ablation, whereby myelinated axon number is restored to control levels. Remarkably, we find that myelin sheaths of normal length and thickness are regenerated during this time. Interestingly, we find that axons grow significantly in caliber during this period of remyelination. This suggests the possibility that the active growth of axons may stimulate the regeneration of myelin sheaths of normal dimensions.

## Introduction

Myelin is essential for life-long nervous system health and function. This is illustrated by the fact that disruption to myelin of the central nervous system (CNS) is associated with numerous neurodegenerative [1], neurodevelopmental [2,3] and neuropsychiatric disorders [4]. Indeed, loss of CNS myelin (demyelination) has long been known to be prevalent in multiple sclerosis (MS) [5], and it is now thought that long-term demyelination contributes to the axonal degeneration [6] associated with progressive stages of the disease [7,8]. Fortunately, endogenous regeneration of myelin (remyelination) can occur following CNS demyelination in both animal models (e.g. [9–11]) and disease (e.g.[12]). Principal to the capacity for myelin regeneration is the presence of oligodendrocyte progenitor cells (OPCs) in the CNS throughout adult life [13]. OPCs have the capacity to generate myelinating oligodendrocytes from development well in to adulthood in the healthy brain [14–16], and also in response to demyelination [17,18].

Unfortunately, the regenerative response of remyelination is typically insufficient in MS, and typically fails with increasing age [19–22]. Demyelination occurs secondarily following spinal cord injuries [23], and although remyelination can occur on spared axons, it is insufficient to fully restore function [24]. In recent years a number of intrinsic and extrinsic factors have been identified that can influence remyelination efficiency, offering the possibility to use this knowledge to develop therapeutic interventions [25]. However, the pathologies observed following demyelination are complex [26,27], posing a range of challenges for therapeutic targeting. For example, in MS some demyelinated lesions have no OPCs at all [28], others have abundant OPCs that fail to differentiate [29–31], and others differentiated oligodendrocytes that fail to myelinate [32]. In contrast, other lesions and even individual patients appear to undergo relatively robust remyelination [33,34]. Furthermore, remyelination offering hope that this endogenous regenerative process can be enhanced. However, even in areas of robust myelin regeneration, the process of remyelination is imperfect, with remyelinated lesions typically characterised by myelin sheaths that are shorter and thinner than normal [12]. Mirroring observations in disease states, remyelination observed in animal models also typically culminates in the formation of myelin sheaths that are shorter [10] and thinner [9,11] than seen around unaffected axons of the same size. It remains almost entirely unclear why myelin sheaths made during remyelination are abnormally short and thin [35]. Our lack of knowledge in part reflects the fact that research to date has focussed primarily on promoting oligodendrocyte differentiation, which is seen as a major bottleneck to remyelination [6,36]. Although great progress has been made towards identifying strategies to promote oligodendrocyte differentiation (e.g. [37–43]), our understanding of what controls myelin sheath size remains relatively limited. The precise number, distribution, length, and thickness of myelin sheaths along axons all have predictable effects on conduction speed and thus timing of neuronal communication [44–49]. Indeed, regulation of precise conduction times by fine-tuning myelin sheath parameters may represent a fundamental form of functional plasticity [50]. Although the regeneration of shorter and thinner myelin sheaths can restore basic conduction [51], regeneration of myelin fine-tuned to mediate optimal circuit function is likely to require restoration of myelin sheaths of the correct parameters [52,53]. Therefore, it is a reasonable supposition that regeneration of normal myelin sheaths may be required to restore cognitive functions impaired in diseases of myelin, including MS [54].

We developed a transgenic-based drug-inducible model of oligodendrocyte ablation, with subsequent demyelination and remyelination in the larval zebrafish (*Danio rerio*). Our aim was to take advantage of the supreme qualities of zebrafish larvae for imaging myelinated axons [55] to follow the response to demyelination of specific axons over time. We chose to study the response to demyelination of reticulospinal axons in the zebrafish CNS, as reticulospinal axons represent a discrete, well-defined identifiable population that are robustly myelinated along their length by just 5 days post-fertilisation (dpf) [56]. Using the nitroreductase-metronidaxole system [57], similar to approaches recently described in both zebrafish and *Xenopus laevis* [58–61], we were able to ablate 2/3rds of myelinating oligodendrocytes in the larval zebrafish spinal cord at early larval stages. This ablation of oligodendrocytes was characterised over the subsequent week by widespread myelin vacuolation, axonal demyelination, and a macrophage-microglial inflammatory response. We find that during the week following cell ablation, oligodendrocyte and myelinated axon number increased to control levels, indicating rapid regeneration of myelin. Furthermore, and somewhat surprisingly, myelin sheaths of similar length and thickness to controls were regenerated during this time. Interestingly we noted that this remyelination occurred during a period when reticulospinal axons grew significantly in caliber. Given the recent indication that axon caliber is a potent regulator of CNS myelination [62–65], we suggest that regeneration of myelin sheaths of the correct size may be driven by ongoing growth of axons in caliber.

## Materials and methods

### Maintenance of adult zebrafish

Adult zebrafish were maintained in a dedicated aquatic facility at 28.5°C and under a 14/10 hour light/dark cycle. All animal studies were carried out with approval from the UK Home Office and according to its regulations, under project licenses 60/ 8436 and 70/8436. The project was approved by the University of Edinburgh Institutional Animal Care and Use Committee.

The following transgenic zebrafish lines were used in this study: Tg(mbp:mCherry-NTR) (generated for this study), Tg(mbp:EGFP) (Almeida et al. 2011), Tg(mbp:EGFP-CAAX) (Almeida et al. 2011) and Tg(mpeg:GFP) [66]. The Tg(mbp:mCherry-NTR) and Tg(mbp:nls-GFP) lines used were generated as part of this study.

### Generation of the mbp:mCherry-NTR and mbp:nls-GFP transgenic lines

We used the tol2kit [63] to construct the plasmids required create the Tg(mbp:mCherry-NTR) and Tg(mbp:nls-GFP lines. To generate mbp:mCherry-NTR we first PCR amplified the nitroreductase sequence from a template plasmid (HindIII_NTR_forward GACAAGCTTATGGATATCATTTCTGTCGCC and XhoI_NTR_reverse CAGACTCGAGGTTACACTTCGGTTAAGGTG) and cloned it into pCS2+ using HindIII/XhoI. This was used as template to PCR amplify NTR-polyA (attB2-NTR-F: GGGGACAGCTTTCTTGTACAAAGTGGACATGGATATCATTTCTGTCGCC and attB3-SV40pA R: GGGGACAACTTTGTATAATAAAGTTGAAAAAACCTCCCACACCTCCC). The PCR product was recombined with pDONR_P2P3R using BP clonase enzyme to generate p3E_NTR-pA. The mbp:mCherry-NTR plasmid was generated by recombining the above described 3’ entry clone containing the NTR sequence, a 5’ entry clone containing the regulatory sequence for the myelin basic protein (mbp) [65] and the pME_mCherry fluorescent middle entry clone with the pDEST_Tol2_PA2 destination vector. This ligation was done using LR clonase II Plus (Invitrogen). The mbp:mCherry-NTR plasmid thus generated was then injected into fertilised wild type zebrafish eggs at the 1–8 cell stage to create embryos that expressed the transgene mosaically. Larvae that showed robust mosaic expression of the transgene at 5 days post fertilization (dpf) were raised to adulthood, from which F1 founder Tg(mbp:mCherry-NTR) stable transgenic animals were identified. All experiments were performed F2 and subsequent generations of this line. To generate the mbp:nls-GFP, we recombined the same 5’ entry clone containing mbp regulatory sequence with pME_nls-GFP, which is part of the tol2kit, and generated stable lines as above for Tg(mbp:mCherry-NTR). In Figs and legends, “Tg” before the line name denotes a stable transgenic line, whereas a transgene name indicates that a plasmid was injected, resulting in mosaic expression.

### DNA microinjections

For mosaic labelling, fertilised eggs were microinjected with the appropriate DNA construct at the 1–8 cell stage. Eggs were injected with 1nl of a solution containing 5–20 ng/μl plasmid DNA, 12–20 ng/μl tol2 transposase mRNA, and 10% phenol red.

### Metronidazole treatment and maintenance of larvae post-treatment

Treatment with metronidazole (Mtz) was started at 5dpf. Larvae were placed in 50ml petri dishes containing either 5mM metronidazole (Mtz), 1% DMSO solution or only 1% DMSO in embryo medium, and kept in a 28°C incubator for 48h. After 24h of treatment, the solutions were refreshed. At the end of the 48h treatment, the larvae were transferred into one-litre tanks, and maintained there up to 16 days (16 days post-treatment, dpt).

### Live imaging

For live imaging, larvae were anaesthetised in tricaine as above, and embedded in 1.3% low melting point agarose (Invitrogen). Confocal images were acquired using Zeiss LSM 710 or 780 confocal microscopes with a 20X lens, with the exception of live imaging of single oligodendrocytes at 23dpf, which was performed on a Zeiss LSM 880 Airyscan microscope with a 10X lens (subjected to a post-magnification zoom of 1.8 and a 3D post-acquisition deconvolution using the Wiener filter). All live images are oriented as lateral views of the spinal cord, with anterior towards the left and dorsal towards the top of the image.

### Preparation of samples for transmission electron microscopy (TEM)

Preparation of zebrafish tissue for transmission electron microscopy was performed according to the previously published protocol (Czopka and Lyons, 2011). Silver sections (70nm in thickness) were cut at the level of somite 16 using a Reichert Jung Ultracut microtome (Leica) and a Diatome diamond knife. TEM imaging was performed at the University of Edinburgh Electron Microscope Facility using a Phillips CM120 Biotwin transmission electron microscope. Image magnification ranged from 1600x to 4800x.

Electron microscopy data is presented as images of whole hemi-spinal cords, ventral tracts of one hemisphere (ventral hemi-spinal cords) or individual tiles captured from ventral tracts. In order to create panoramic views, individual electron micrograph tiles were aligned using the automated photomerge tool in Adobe Photoshop CC. Quantification from electron micrographs was performed using Fiji.

### Preparation of cryosections

Larvae were fixed with 4% paraformaldehyde in PBS overnight at 4°C. They were then washed with PBS, and transferred to a 30% sucrose solution in PBS. They were aligned in silicone moulds containing optimal cutting temperature (OTC) medium (Scigen) and immediately snap frozen in 2-methylbutane (Sigma) immersed in liquid nitrogen. Cutting was performed using a Leica CM3050S cryostat. 14μm thick sections were cut starting at somite 5, located just posterior to the swim bladder.

### Hoechst staining, imaging, and analysis of cryosections

Cryosections were incubated with 1 mg/ml (1:1000) Hoechst (Thermo Scientific) for 10 min and subsequently washed with 0.02% Triton-X (Arcos) in PBS and finally with PBS (Lonza). They were then imaged on Zeiss LSM 710 or 780 microscopes with a 40x oil immersion lens, using a post-magnification zoom of 2. The 405nm channel was used to visualise the Hoechst staining, and the 568nm channel to visualise the endogenous mCherry signal from the mbp:mCherry-NTR transgene. Four sections were imaged per larva.

### Acridine orange staining

For labelling cells undergoing cell death *in vivo*, living larvae were incubated with 3μg/μl acridine orange (Sigma) at 28°C for ten minutes [67]. The 488nm laser was used to excite the samples.

### Statistical analysis

All graph-making and statistical tests were carried out using GraphPad Prism. All data were averaged per biological replicate (N represents number of larvae). Unless otherwise noted, data was assumed to be normally distributed and was compared between vehicle and drug-treated groups using a two-tailed unpaired Student’s t-test, considering p < 0.05 as a significant result. Where multiple variables were considered, a two-way ANOVA was used to assess significance. Throughout the Figs, error bars illustrated mean ± standard deviation (SD). Statistical significance is indicated as follows: * p <0.05, ** p <0.01, *** p <0.001, **** p < 0.0001.

## Results

### Transgenic system ablates two thirds of oligodendrocytes in larval zebrafish

**Fig 1:**
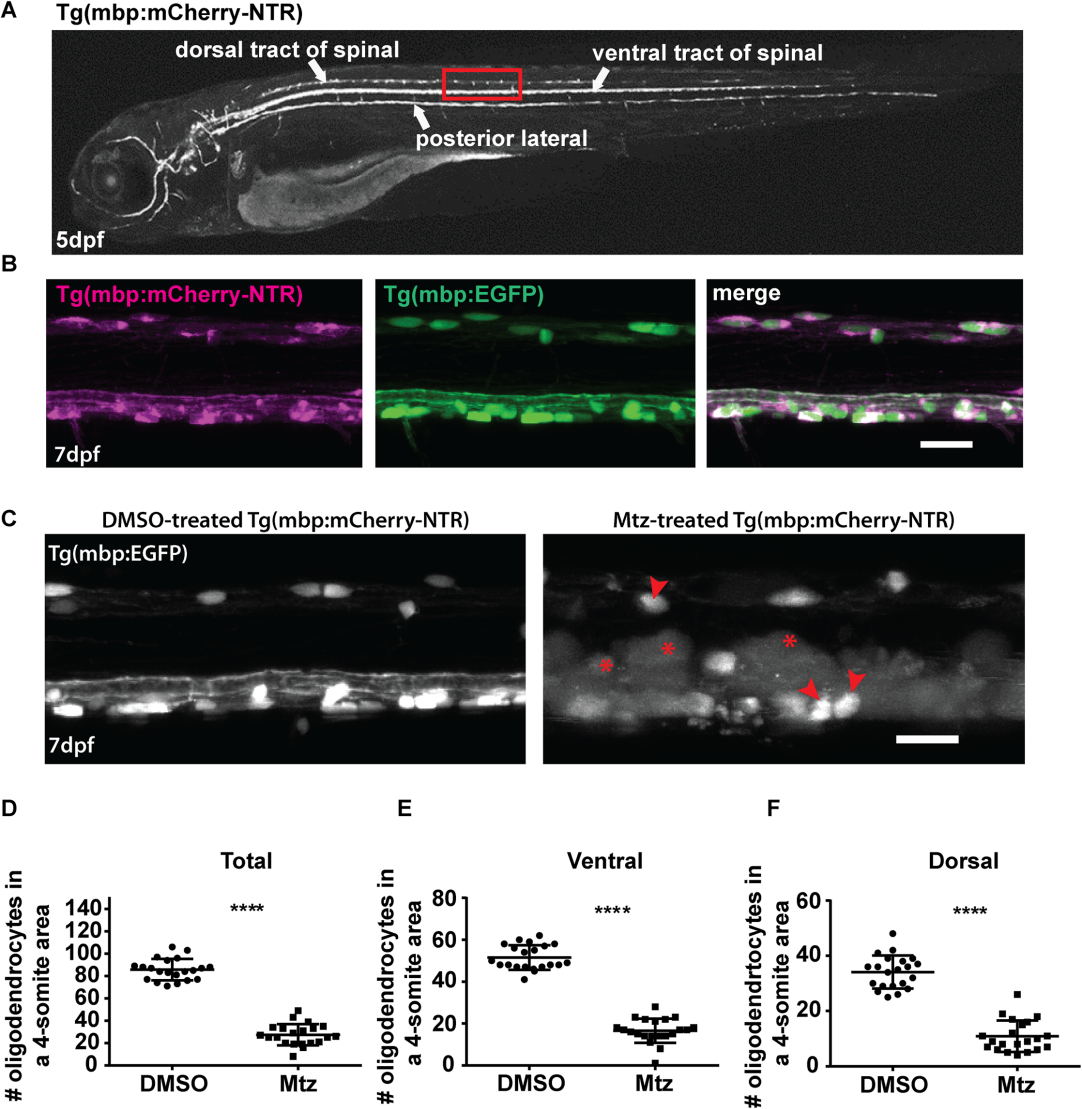
Oligodendrocyte ablation using the Tg(mbp:mCherry-NTR) model. **A**. Top panel: brightfield image of a Tg(mbp:mCherry-NTR) larva at 5dpf. Bottom panel: fluorescent mage showing the expression of the mbp:mCherry-NTR transgene in the larva. The dorsal and ventral tracts of the spinal cord and the posterior lateral line are indicated. The red box outlines the region that is shown in higher magnification in **B** (in another animal). **B**. Lateral views of the spinal cord of double transgenic Tg(mbp:mCherry-NTR);Tg(mbp:EGFP) larvae at 7dpf, showing complete overlap of the mCherry and GFP channels. Scale bar: 20μm.**C**. Oligodendrocyte ablation in Tg(mbp:mCherry-NTR) animals following two-day treatment with Mtz. Red arrowheads indicate unaffected oligodendrocytes and red asterisks indicate vacuolated structures. Scale bar: 20μm.**D-F**. Quantification of oligodendrocyte numbers from a four-somite stretch of the spinal cord in the entire region (**D**) ventral (**E**) and dorsal (**F**) tracts of DMSO and Mtz-treated larvae at 7dpf (immediately following treatment). Overall oligodendrocyte number (dorsal and ventral combined), controls: 85.71 ± 2.1 vs treated: 27.48 ± 2.1 (68% reduction, p < 0.0001). n = 21. In the ventral tract, controls: 51.57 ± 1.3 vs treated: 16.57 ± 1.3 (68% reduction, p < 0.0001). In the dorsal tract, mean in controls: 34.14 ±1.3 vs treated: 10.9 ± 1.2 (68% reduction, p < 0.0001).

To study the cellular mechanisms of demyelination and remyelination over time, we developed a transgenic zebrafish model in which we could conditionally ablate myelinating oligodendrocytes. To do so, we employed the nitroreductase-metronidazole system. Briefly, in this system, the bacterial enzyme nitroreductase is expressed specifically in cell types of interest. This expression in and of itself has no effect on the cells, but upon treatment with the prodrug metronidazole (Mtz), the nitroreductase metabolises metronidazole, yielding a toxic byproduct which causes DNA interstrand cross-linking and subsequent death of the cells expressing the enzyme [57]. Bystander effects on non-expressing cells have not been reported [57]. We expressed a fusion protein of nitroreductase (NTR) and the fluorescent reporter mCherry in myelinating glial cells using the previously published zebrafish myelin basic protein (mbp) promoter [65], and generated a stable transgenic line in which all myelinating glial cells expressed the fusion protein (Fig 1A–1C, Materials and Methods). To determine how faithful expression of the NTR enzyme was in myelinating oligodendrocytes, we crossed Tg(mbp:mCherry-NTR) with the previously characterised stable transgenic line Tg(mbp:GFP). We found that essentially all (99% ± 1.30%) GFP expressing cells expressed the nitroreductase-mCherry transgene (Fig 1B).

We have previously shown that reticulospinal neurons, which project large caliber axons along the length of the ventral spinal cord, are robustly myelinated by 5 days post fertilisation [56]. Therefore, we reasoned that these axons would be a tractable system in which to study the response to demyelination of a defined axonal population. We treated Tg(mbp:mCherry-NTR) fish with 5mM Mtz from 5dpf to 7dpf, and found that this lead to a reproducible loss of 2/3rds of oligodendrocytes in the spinal cord (quantified by counting remaining oligodendrocyte cell bodies; Fig 1D). This reduction in cell number was consistent in both the ventral tract, where reticulospinal axons are located, and dorsal tract: at 7dpf, immediately following treatment (0 days post-treatment): mean number of oligodendrocytes in control ventral tracts: 51.57 ± 1.30 vs treated: 16.57 ± 1.30, 68% reduction, p < 0.0001). In control dorsal tracts: 34.14 ± 1.30 vs treated: 10.9 ±1.20, 68% reduction, p < 0.0001. (Fig 1D). Importantly, treating Tg(mbp:nls-GFP) transgenic animals (which do not express NTR) with Mtz did not affect oligodendrocyte number (S1 Fig). Notably, treatment of Tg(mbp:mCherry-NTR) with Mtz also resulted in the appearance of large vacuole-like structures (asterisk) which we will discuss below and see also S2 Fig).

### Oligodendrocyte number recovers to control levels by 16 days post treatment

**Fig 2:**
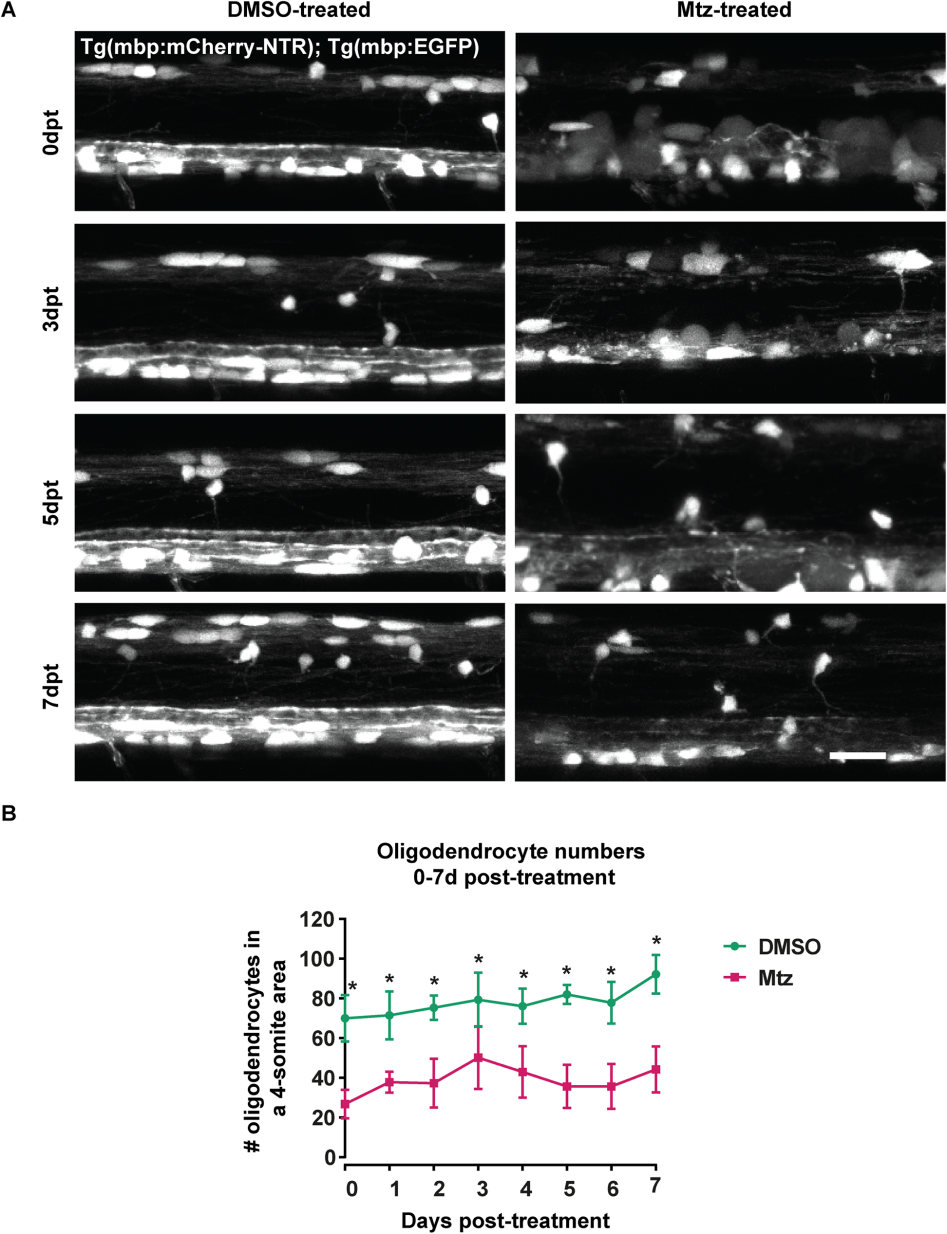
Oligodendrocyte numbers remain low in Mtz-treated Tg(mbp:mCherry-NTR) larvae for 7 days following treatment. **A**. Representative images from the spinal cords of DMSO- and Mtz-treated Tg(mbp:mCherry-NTR) larvae at the time points indicated; GFP channel is shown. **B**. Quantification of oligodendrocyte numbers from a four-somite stretch of the spinal cord. At 0dpt, mean number of oligodendrocytes in controls: 69.94 ± 11.64 vs treated: 26.76 ± 7.20, p < 0.0001. At 1dpt, controls: 71.43 ± 12.09 vs treated: 37.75 ± 5.34, p < 0.0001. At 2pt, controls: 75.29 ± 6.16 vs treated: 37.22 ± 12.28, p < 0.0001. At 3dpt, controls: 79.29 ± 13.71 vs treated: 50.13 ± 15.78, p < 0.0001. At 4dpt, controls: 76.08 ± 8.92 vs treated: 42.95, p < 0.0001. At 5dpt, controls: 81.92 vs treated: 35.60 ± 10.88, p < 0.0001. At 6dpt, controls: 77.78 ± 10.54 vs treated: 35.64 ± in 11.3, p < 0.0001. At 7dpt, controls: 92.15 ± 9.76 vs treated: 44.25 ± 11.57, p < 0.0001. Statistical significances were determined by multiple t tests per row, without assuming equal standard deviations (Holm-Sidak method). n = no less than 7. Scale bar: 20μm.

To understand how the oligodendrocyte lineage responds to the reduction in cell number over time, we performed a time-course analysis of mbp:GFP expressing cells in control and Mtz treated Tg(mbp:mCherry-NTR) animals. We first quantified cell number in the spinal cord in live Tg(mbp:GFP) animals [65] for the first seven days post Mtz treatment (7-14dpf/ 0-7dpt), as per our initial analysis above. Analysing a sample region of four somites per spinal cord, we found a mean total (dorsal and ventral) of 69.94 ± 11.64 oligodendrocytes at 0dpt in controls, which increased to 92.15 ± 9.76 by 7dpt (Fig 2). In contrast, the total number of oligodendrocytes in Mtz-treated animals was reduced to a mean of 26.76 ± 7.20 at 0dpt, and over the following week, increased to 44.25 ± 11.57 by 7dpt (Fig 2).

**Fig 3:**
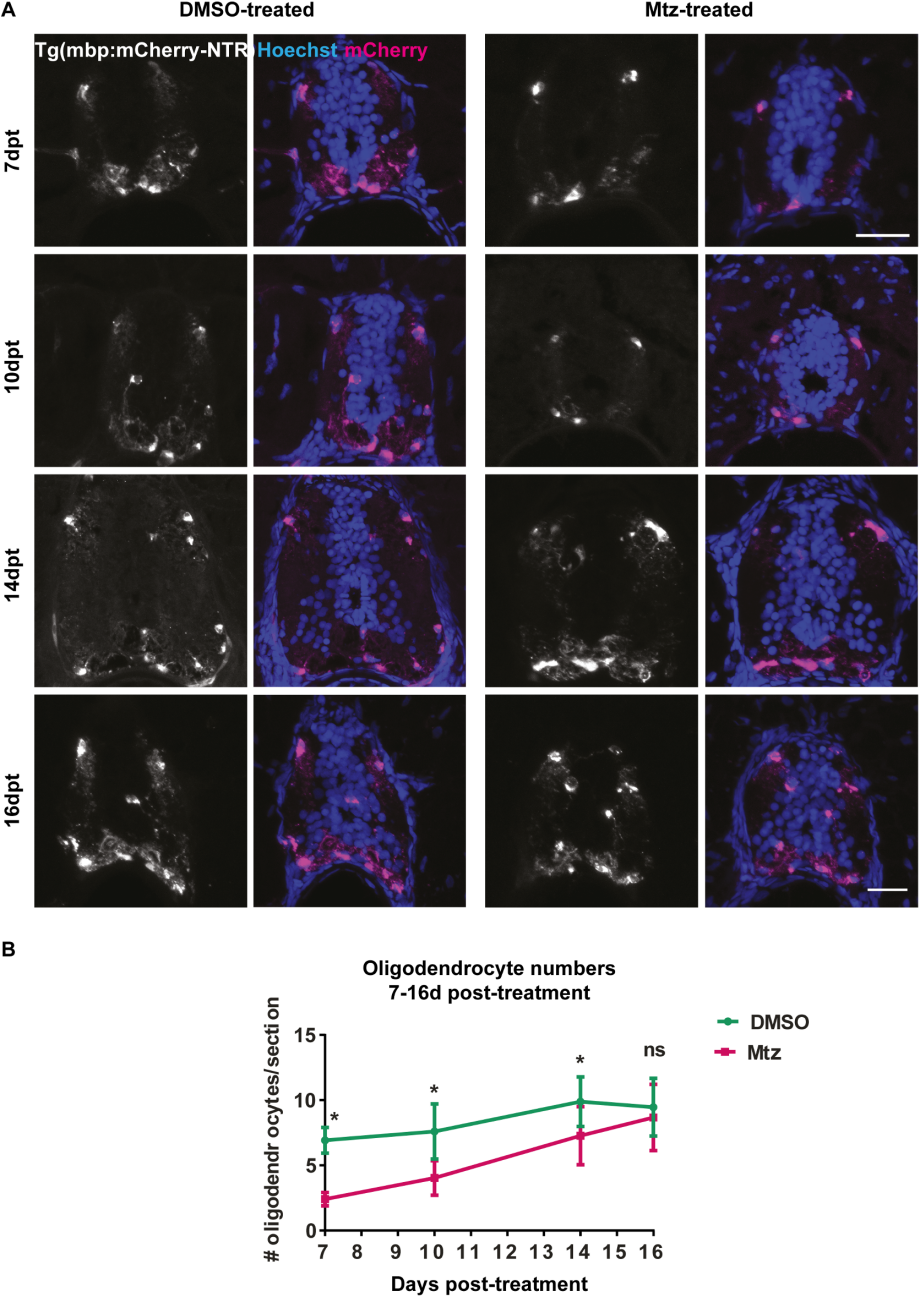
Oligodendrocyte numbers are restored to control levels by 16 days post-treatment. **A.** Representative images of transversely cut cryosections of spinal cords stained with Hoechst and showing endogenous expression of the mbp:mCherry-NTR transgene. Time points from 7d post-treatment to 16d post-treatment are shown, as indicated. Yellow arrowheads point to examples of double positive cells that were counted as oligodendrocytes.**B**. Quantification of oligodendrocyte numbers per section. At 7dpt, mean in controls: 6.9 ± 0.44 vs treated: 2.4 ± 0.23 in Mtz-treated sections, p < 0.0001. At 10dpt, controls: 7.6 ± 0.75 vs treated: 4.04 ±0.47, p = 0.0012. At 14dpt, controls: 9.88 ± 0.6 vs treated: 7.3 ± 0.67, p = 0.0095. At 16dpt, controls: 9.46 ± 2.21 vs treated: 8.57 ± 2.58, p = 0.260. All significances were obtained using multiple t tests per row (Holm-Sidak method). n = no less than 5. Scale bars: 20μm.

Larger animals >2 weeks old (7dpt) are more difficult to image live in wholemount preparations. Therefore, to quantify oligodendrocyte number from 7dpt onwards, we cut transverse sections through the spinal cord of control and Mtz treated Tg(mbp:mCherry-NTR) animals. This analysis showed that the significant difference in oligodendrocyte numbers between control and Mtz-treated animals, observed at 7dpt (65% reduction in cell number), was no longer present at 16dpt due to the large linear increase in oligodendrocyte number in MTZ-treated animals observed over this time (Mean number of oligodendrocytes per section at 7dpt: in controls: 6.9 ± 0.44 vs treated: 2.4 ± 0.23, p < 0.0001. At 16dpt, in controls: 9.46 ± 2.21 vs treated: 8.57 ± 2.58, p = 0.260) (Fig 3). Thus oligodendrocyte number is restored to control levels by 16dpt.

### Oligodendrocyte ablation results in myelin vacuolation and widespread tissue disruption

**Fig 4:**
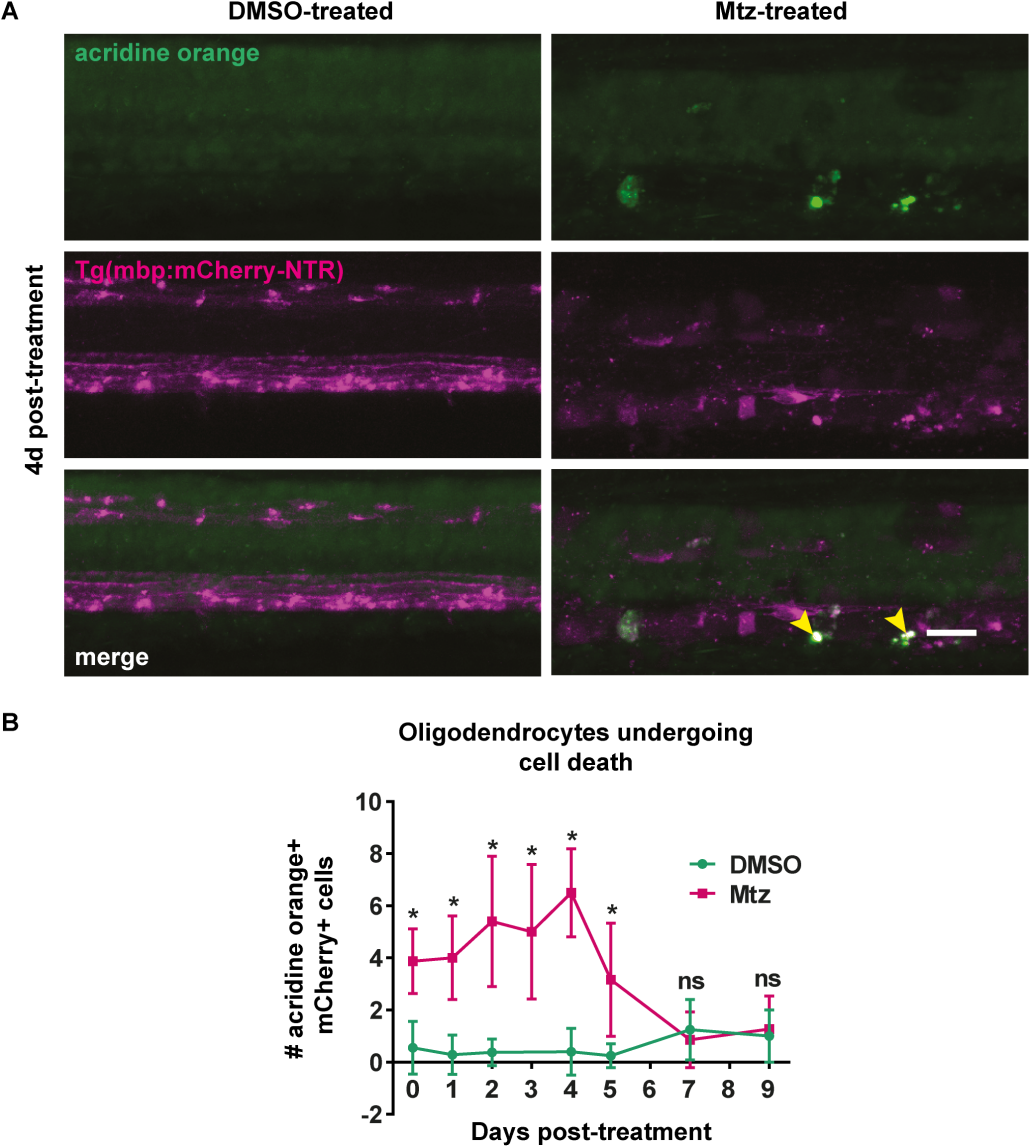
Acridine orange staining reveals oligodendrocytes undergoing cell death following treatment with Mtz. **A**. Representative images of spinal cords of DMSO- or Mtz-treated Tg(mbp:mCherry-NTR) larvae at 4dpt. Acridine orange labels cells undergoing cell death in green. In Mtz-treated animals several bright green puncta can be seen, and these colocalise with mCherry (yellow arrowheads). **B**. Quantification of cells that are positive for both mCherry and acridine orange, per four-somite stretch of spinal cord. At 0dpt, control mean: 0.56 ± 1.01 vs treated: 3.88 ± 1.25, p < 0.0001. The number of AO+ cells peaks at 4dpt, when the mean in controls was: 0.4 ± 0.89, vs treated: 6.5 ± 1.69, p < 0.0001. This then reduced so that at 7dpt, controls: 1.25 ± 1.16 vs treated: 0.86 ± 1.07, p = 0.510. All significances were obtained using multiple t tests per row (Holm-Sidak method). n = no less than 8. Scale bar: 20μm.

During the course of our analysis of Tg(mbp:mCherry-NTR) animals by brightfield and fluorescent reporter-based imaging during the first week following treatment, we noted the appearance of striking, large vacuolated structures in Mtz treated animals (S2 Fig). These structures were visible throughout Mtz treatment, and remained prominent until 9dpt, by which time they were no longer detectible (in the majority of animals). Analysis of mbp:GFP-CAAX, which allows visualisation of myelin membrane, suggested that these structures represented vacuolated myelin (S2 Fig), similar to what has previously been reported in mammalian models of oligodendrocyte ablation [68–71]. Perhaps surprisingly, during this time, we do not observe any loss of axons (see further discussion below). To investigate whether ablated oligodendrocytes were undergoing cell death, we used the vital dye acridine orange (AO) to label fragmented nucleic acids [72], and which we have previously used to quantify cell death in the larval zebrafish [67]. We observed an increase in AO +ve labelled cells in Tg(mbp:mCherry-NTR) Mtz treated animals compared to controls, peaking at 4dpt (control mean: 0.4 ± 0.89, vs treated: 6.5 ± 1.69, p < 0.0001) (Fig 4).

### Innate immune response to oligodendrocyte ablation

**Fig 5:**
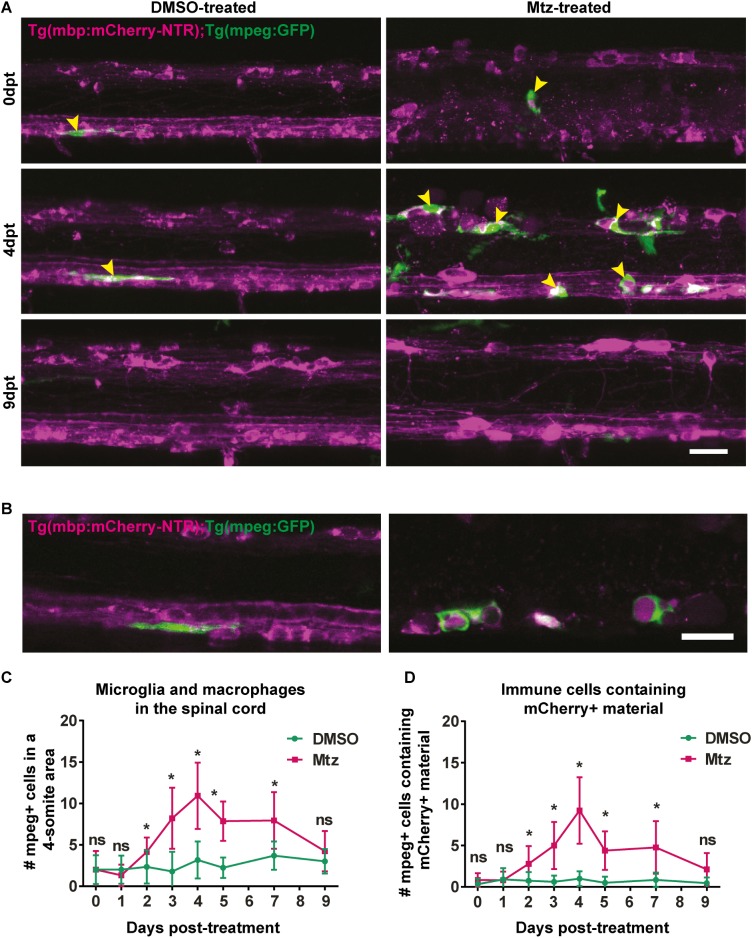
Macrophage and microglia response to oligodendrocyte ablation. **A**. Representative images of spinal cords of DMSO- or Mtz-treated Tg(mbp:mCherry-NTR) larvae, Green cells are mpeg-expressing microglia and macrophages. Yellow arrowheads indicate examples of mpeg+ cells in contact with the spinal cord. **B.** Single z-plane images from DMSO- or Mtz-treated Tg(mbp:mCherry-NTR) larvae, Green cells are mpeg-expressing microglia and macrophages and can be seen to surround mCherry positive structures in Mtz-treated animals. **C**. Quantification of mpeg+ cells in contact with the spinal cord (from a four-somite stretch of the spinal cord) At 0dpt, mean in controls: 2.0 ± 1.73 vs treated: 2.0 ± 2.26, p > 0.9999. At 1dpt, controls: 2.0 ± 1.71 vs treated: 1.31 ± 1.3, p = 0.237. At 2dpt, controls: 2.33 ± 2.02 vs treated: 4.14 ± 1.75, p = 0.022. At 3dpt, controls: 1.79 ± 2.39 vs treated: 8.2 ± 3.68, p < 0.0001). At 4dpt (controls: 3.17 ± 2.22 vs treated: 10.92 ± 4.01, p = 0.00040). At 5dpt, controls: 2.23 ± 1.24 vs treated: 7.84 ± 2.38, p < 0.0001. At 7dpt, controls: 3.69 ± 1.70 vs treated: 7.94 ± 3.42, p = 0.0003. At 9dpt, there is no significant difference between control and Mtz-treated animals (controls: 3.0 ± 1.48 vs treated: 4.22 ± 2.44, p = 0.18). Statistical significance was determined using multiple t tests per row, with a Holm-Sidak method. n = no less than 9. Scale bar: 20μm. **D.** Quantification of mpeg mpeg+ cells containing mCherry+ material in control Mtz-treated animals.At 1dpt, controls: 0.92 ± 1.38 vs treated: 0.81 ± 1.05 in Mtz-treated animals (p = 0.822). At 2dpt, controls: 0.75 ± 1.06 vs treated: 2.79 ± 2.16 (p = 0.0066).At 3dpt: controls: 0.62 ± 0.77 vs treated: 5.0 ± 2.85 (p < 0.0001). At 4dpt: controls: 1 ± 0.89 vs treated: 9.23 ± 4.02 in Mtz-treated animals (p = 0.00014). At 5dpt: controls: 0.50 ± 0.76 vs treated: 4.39 ± 2.33 (p < 0.0001). At 7dpt: controls: 0.85 ± 0.90 vs treated: 4.75 ± 3.17 cells in Mtz-treated animals (p = 0.00018). At 9dpt: controls: 0.45 ± 0.69 cells in controls and 2.11 ± 1.96 cells in treated animals (p = 0.017). This was not deemed significant by the Holm-Sidak method. Statistical significance was determined using multiple t tests per row, with a Holm-Sidak method. n = no less than 9. Scale bar: 20μm.

The appearance of apoptotic carcasses and abundant myelin debris prompted us to ask whether this elicited an immune response. We therefore performed a time-course analysis of the transgenic reporter line Tg(mpeg:GFP) [66] crossed into Tg(mbp:mCherry-NTR) line, to assess the response of macrophages/ microglia to oligodendrocyte ablation. We found a sharp rise in the number of mpeg:GFP-expressing cells in treated animals compared to controls from 3dpt, peaking at 4dpt (control mean: 3.17 ± 2.22 vs treated: 10.92 ± 4.01, p = 0.00040) and subsiding soon after the first week post-treatment, so that by 9dpt, there was no significant difference between control and Mtz-treated animals (mean in control: 3.0 ± 1.48 vs treated: 4.22 ± 2.44, p = 0.18) (Fig 5A and 5C). Similarly, further analysis showed a corresponding increase in the number of mpeg expressing cells that contained NTR-mCherry protein (Fig 5B and 5D), suggesting that these cells actively engulfed myelin debris. Together these analyses indicate that from two to nine days following cell ablation, the immune system responds and actively phagocytoses myelin debris, which is no longer apparent after this time.

Interestingly, oligodendrocyte number only increases after this immune response has resolved, suggesting that immune-mediated clearance of damaged tissue may be required for the regeneration of oligodendrocytes to begin, as indicated in rodent models of demyelination [73].

### Oligodendrocyte ablation leads to extensive demyelination

**Fig 6:**
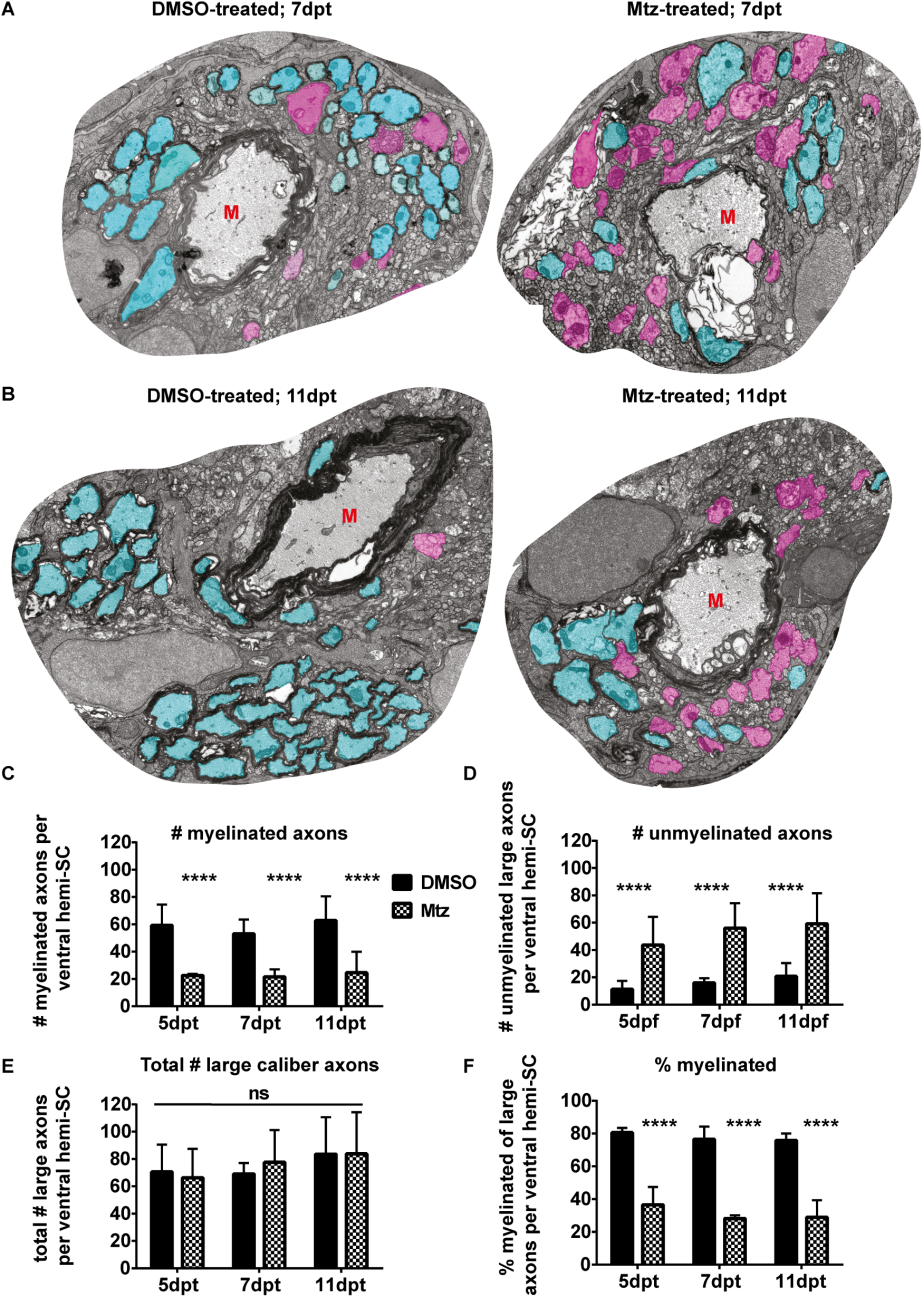
Oligodendrocyte ablation results in extensive demyelination of reticulospinal axons. **A-B**. Representative electron micrographs of ventral hemi-spinal cords of DMSO- or Mtz-treated Tg(mbp:mCherry-NTR) larvae at 7dpt or 11dpt as indicated. Myelinated axons are shaded in turquoise and non-myelinated axons in pink. Red letters M indicate Mauthner axons. Scale bar: 1μm. **C.** Quantification of the number of myelinated axons per ventral hemi-spinal cord at 5, 7 and 11d following withdrawal of Mtz-treatment. At 5dpt, mean in controls: 59.25 ± 15.22 vs treated: 22.5 ± 1.29. At 7dpt, controls: 53.0 ± 10.42 vs treated: mean 21.5 ± 5.51. At 11dpt, controls: 62.75 ± 17.73 vs treated: 24.5 ± 15.46. A two-way ANOVA found a significant main effect of treatment condition (p < 0.0001) but a non-significant main effect of time point (p = 0.597) and non-significant interaction (p = 0.851). **D**. Quantification of the number of unmyelinated axons with a perimeter over 1μm. At 5dpt, mean in controls: 11.25 ± 6.24 vs treated: 43.75 ± 20.52. At 7dpt, mean in controls: 16.0 ± 3.46 vs treated: 56.0 ± 18.24. At 11dpt, mean in controls: 20.75 ± 9.81 vs treated: 59.25 ± 22.43. A two-way ANOVA found a significant main effect of treatment condition (p < 0.0001) but a non-significant main effect of time point (p = 0.274) and a non-significant interaction (p = 0.875). **E**. Quantification of the total number of axons with a perimeter over 1μm per ventral hemi-spinal cord. At 5dpt, mean in controls: 70.5 ± 20.04 vs treated: 66.25 ± 21.23. At 7dpt, controls: 69.0 ± 8.04 vs treated: 77.5 ± 23.70. At 11dpt, controls: 83.5 ± 27.06 vs treated: 83.75 ± 30.51. A two-way ANOVA found a non-significant main effect of treatment condition (p = 0.874) as well as non-significant main effect of time point (p = 0.414) and a non-significant interaction (p = 0.854). **F**. Quantification of the percentage of myelinated axons. At 5dpt, mean percentage of myelinated axons in controls: 80.5% ± 2.89% vs treated: 36.5% ± 10.85%. At 7dpt, controls: 76.5% ± 7.69% vs treated: 28.13 ± 1.93%. At 11dpt, controls: 75.75% ± 4.19% vs treated: 28.88% ± 10.43%. A two-way ANOVA found a significant main effect of treatment condition (p < 0.0001) but a non-significant main effect of time point (p = 0.172) and a non-significant interaction (p = 0.830). n = 4.

In order to determine whether MTZ treatment of Tg(mbp:mCherry-NTR) animals lead to frank demyelination of axons, we performed a time course analyses by transmission electron microscopy (TEM). We first confirmed the widespread disruption to tissue architecture observed by brightfield, fluorescence and electron microscopy (S2 Fig). From 5–11 days post treatment (dpt), we observed clear evidence of demyelination of axons in the ventral spinal cord where reticulospinal axons are located. At 5dpt, we found a mean of 59.25 ± 15.22 myelinated axons in the ventral hemi-spinal cord of controls, but only 22.5 ± 1.29 myelinated axons in Mtz-treated Tg(mbp:mCherry-NTR) animals (p = 0.003). Analysis at 7 and 11dpt revealed that the number of myelinated axons in Mtz treated animals remained lower over this period (7dpt, controls: 53.0 ± 10.42 myelinated axons, vs treated: 21.5 ± 5.51. At 11dpt, controls: 62.75 ± 17.73 vs treated: 24.5 ± 15.46). Over the period from 5dpt to 11dpt, a two-way ANOVA found a significant main effect of treatment condition (p < 0.0001). The main effect of time point and the interaction between time point and treatment were both non-significant (p = 0.597 and p = 0.851, respectively; Fig 6). Importantly, the total number of large-caliber axons (>1 μm perimeter; 0.32 μm diameter) was similar in both control and Mtz-treated larvae at all stages, indicating that acute demyelination did not affect axonal survival and that the Mtz treatment or the transgenic system did not exert a bystander effect on axons (Fig 6). These data indicate a consistent reduction of about 2/3rds of myelinated axons from 5 through 11 days post Mtz-treatment, reflecting the similar reduction observed in oligodendrocyte number over the same period.

### Robust remyelination occurs by 16 days post-treatment

**Fig 7:**
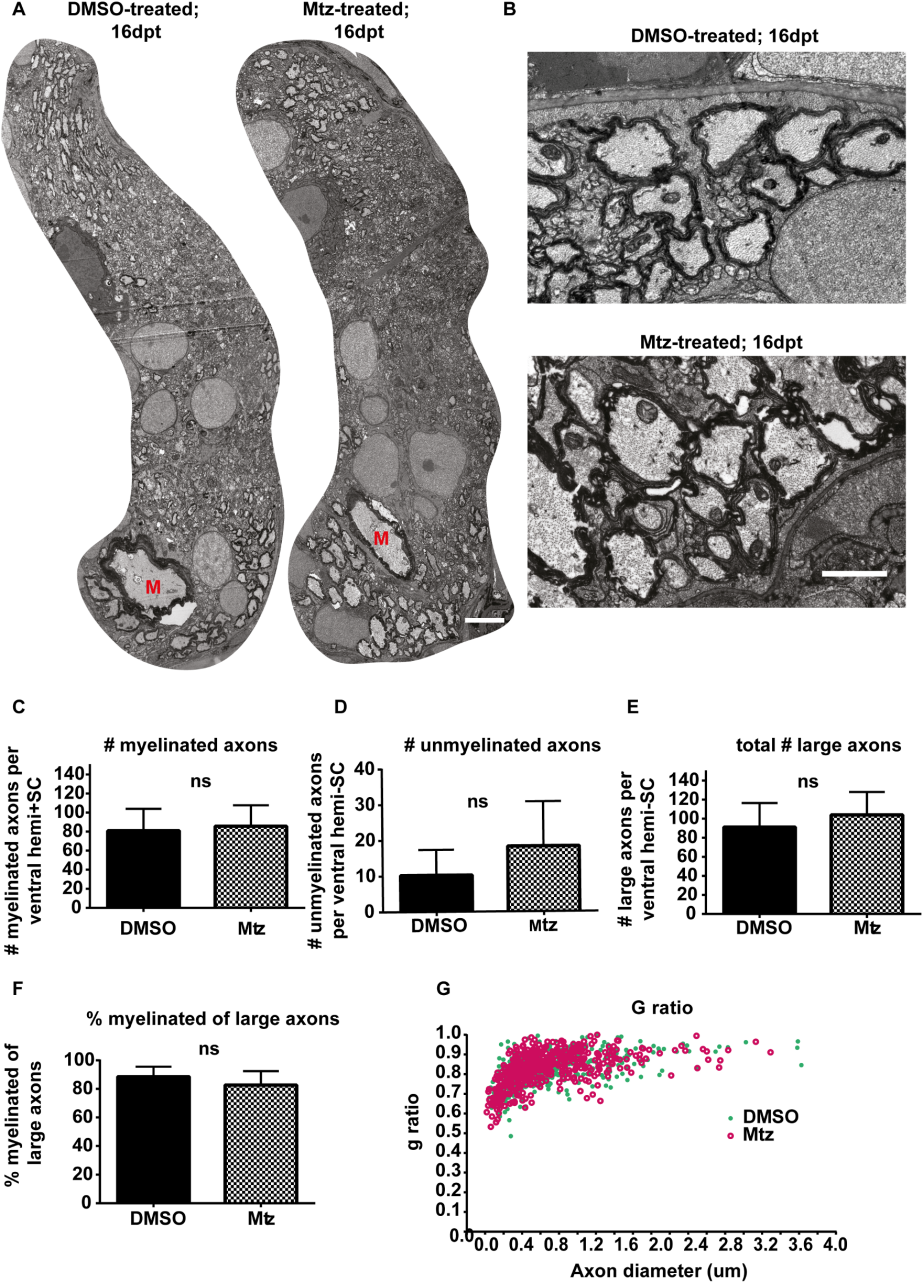
Remyelination occurs by 16 days post-treatment. **A**. Electron micrographs of entire hemispinal cords of DMSO- or Mtz-treated Tg(mbp:mCherry-NTR) larvae). Scale bar: 3μm. **B.** Higher magnification electron micrographs of the ventral spinal cords. Scale bar: 1μm. **C.** Quantification of the numbers of myelinated axons per ventral hemi-spinal cord. Mean in controls: 80.88 ± 23.12 vs treated: 85.71 ± 22.01, p = 0.686. **D**. Quantification of the number of unmyelinated axons < 1μm in perimeter. Mean in controls: 10.25 ± 7.13 vs treated: 18.29 ± 12.47, p = 0.143. **E**. Quantification of the total number of axons with perimeter over 1μm. Mean in controls: 91.13 ± 25.39 vs treated: 104.0 ± 24.04 in Mtz-treated animals, p = 0.334. **F**. Quantification of the percentage of myelinated axons. Mean in controls: 88.75 ± 7.09% vs treated: 82.86 ± 9.72%, p = 0.199. n = 6 for controls, 5 for treated. Significances obtained from two-tailed Student’s t tests.**G**. G ratios plotted against axon diameters show that there is no difference in myelin thickness across axon sizes between control and Mtz-treated animals.

Based on our data that OL number had returned to control levels by 16dpt, we predicted that remyelination of demyelinated axons might occur during this time. To assess the extent of remyelination, we examined animals at 16dpt by TEM in the ventral spinal cord where reticulospinal axons are located. We saw that Tg(mbp:mCherry-NTR) Mtz-treated animals were essentially indistinguishable from controls at this point. We found that the total number of myelinated axons did not differ between treatments (mean in control: 80.88 ± 23.12 vs treated: 85.71 ± 22.01, p = 0.686). Furthermore, the total number of large-caliber axons was comparable in both conditions (control: 91.13 ± 25.39 vs treated: 104.0 ± 24.04, p = 0.334). Therefore, the relative proportion of myelinated axons was consistent between both (control: 88.50% ± 7.03% vs treated: 82.70% ± 9.79%, p = 0.206; Fig 7). This suggests that the increased number of oligodendrocytes made between 7dpt and 16dpt are capable of remyelinating reticulospinal axons in the ventral spinal cord, 2/3rds of which were demyelinated only a few days previously.

**Fig 8:**
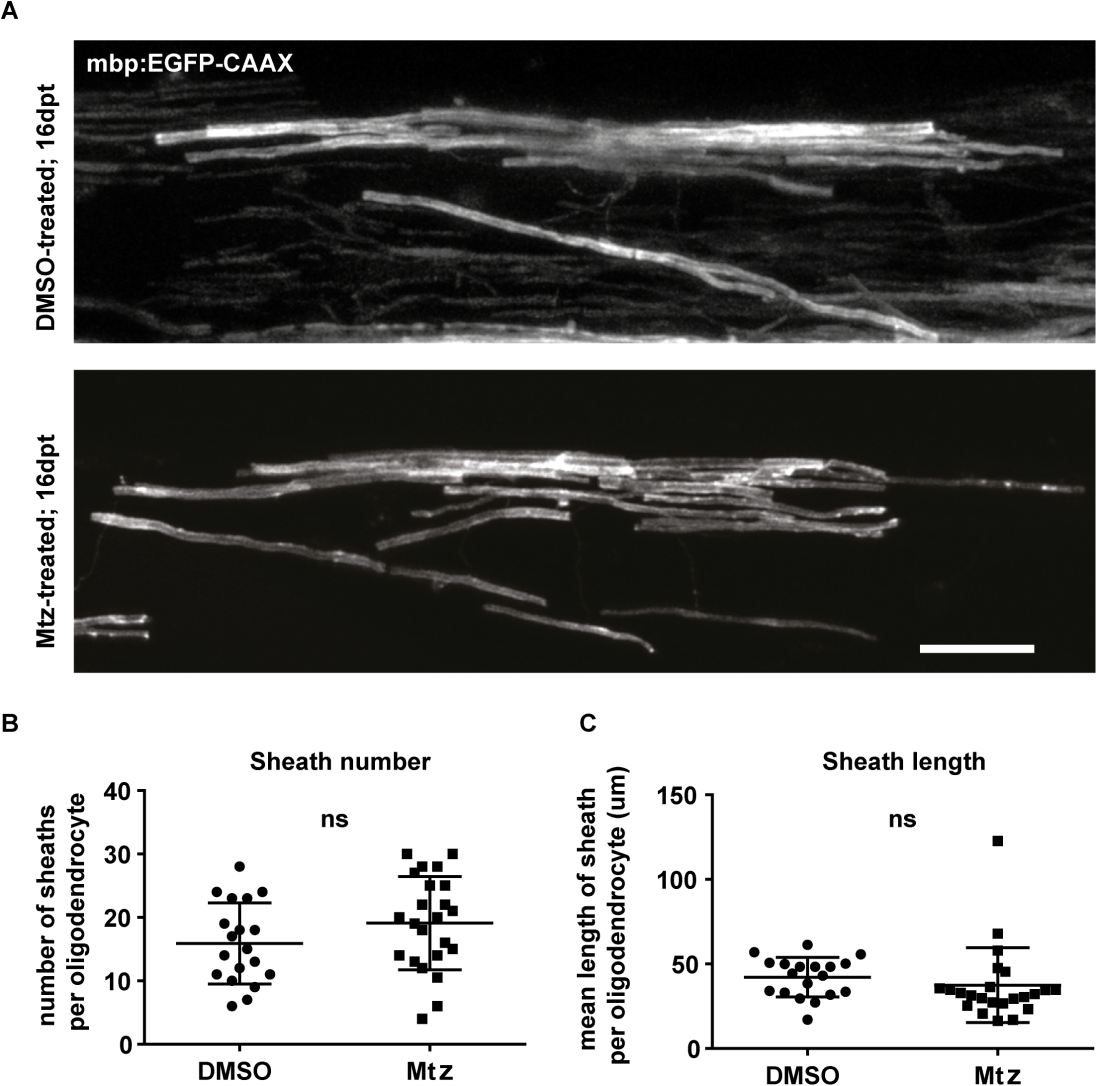
Myelin sheath numbers and lengths per oligodendrocyte are not different between control and treated animals at the remyelinated stage. **A**. Representative images of single oligodendrocytes in DMSO- or Mtz-treated Tg(mbp:mCherry-NTR) fish, labelled with mbp:EGFP-CAAX. Scale bar: 20μm. **B**. Quantification of the mean number of sheaths per oligodendrocyte in control and Mtz-treated animals: in controls: 15.89 ± 6.39, vs treated: 19.11 ± 7.36, p = 0.143 (t test). **C.** Quantification of the mean length of myelin sheath per oligodendrocyte: mean in controls. Mean length of sheath per oligodendrocyte; in controls: 42.21 ± 11.64 vs treated: 37.41 ± 22.14, p = 0.400. n = 19 cells (in 16 animals) for controls, 23 cells (in 19 animals) for treated animals.

Remyelination generally results in the formation of myelin sheaths thinner than normal. Therefore, we measured the g-ratios of axons in Tg(mbp:mCherry-NTR) animals to assess myelin thickness in treated and controls. Interestingly, we found no differences in the g-ratios across treatment (mean g ratio in controls: 0.81 ± 0.042 vs treated: 0.82 ± 0.025, p = 0.671), indicating that in this system remyelination results in the restoration of myelin sheaths with the normal thickness relative to axon caliber (Fig 7F). In addition to the association of remyelination with thinner myelin sheaths, remyelination is also thought to result in myelin sheath that are shorter than normal [12,74]. In order to assess myelin sheath length in Tg(mbp:mCherry-NTR) treated animals at 16dpt, we imaged single oligodendrocytes labelled with the fluorescent reporter mbp:GFP-CAAX to assess myelin sheath number and length per cell. This revealed that both sheath number and, importantly, sheath length were similar in control and treated animals (mean number of sheaths per oligodendrocyte; control: 15.89 ± 6.39, vs treated: 19.11 ± 7.36, p = 0.143. Mean length of sheath per oligodendrocyte: control: 42.21 ± 11.64 vs treated: 37.41 ± 22.14, p = 0.400; Fig 8). This suggests that myelin sheaths of normal length are formed during remyelination in this model. Previous studies have shown contribution of Schwann cells to remyeliantion in the CNS [17]. However, in our analysis of single cell morphology using mbp:GFP-CAAX expressing cells, we did not note any myelinating cells in control or treated animals with a characeteristic Schwann cell morphology, i.e. myelinating just one segment of one axon in our analyses (19 cells in control and 23 in treated animals), suggesting that these cells do not contribute to myelin regeneration in this model. This may be due to the fact that myelinating Schwann cells are also ablated using this transgenic system, given the fact that mbp regulatory sequence drives expression of NTR in both OLs and SCs (Karttunen et al., in prep).

### Axons grow in caliber during the period of remyelination

**Fig 9:**
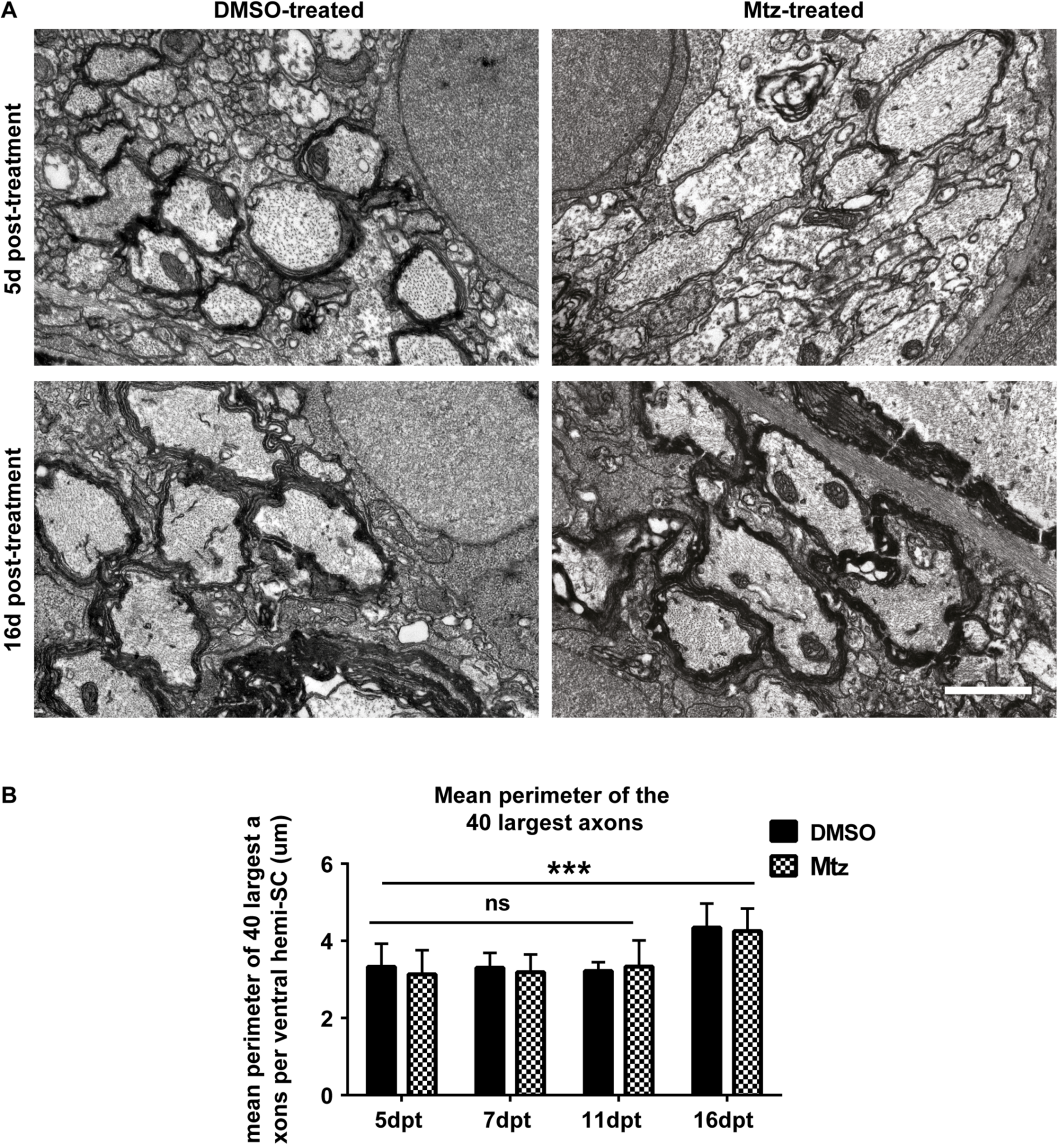
Remyelination coincides with growth of axon caliber between 5dpt and 16dpt. **A**. Top panel: representative electron micrographs from DMSO- and Mtz-treated Tg(mbp:mCherry-NTR) larvae at 5dpt. Bottom panel: same from larvae 16dpt. Scale bar: 1μm. **B**. Quantification of the mean perimeter of the 40 largest axons (whether myelinated or unmyelinated) in control and treated larvae at 5, 7, 11 and 16dpt. At 5dpt, mean perimeter of the 40 largest axons in controls: 3.32 μm ± 0.60 μm and treated 3.13 μm ± 0.62 μm. At 7dpt, controls 3.30μm ± 0.39μm and treated 3.19μm ± 0.46μm. At 11dpt, controls 3.21μm ± 0.23μm and treated 3.33μm ± 0.68μm. At 16dpt, the mean perimeter of the largest 40 axons in controls was 4.34 ± 0.63 in controls and in treated, 4.25 ± 0.59. A two-way ANOVA found a significant main effect of time point (p = 0.0003) but a non-significant main effect of treatment condition (p = 0.707) and a non-significant interaction (p = 0.952).

Given the clear association of axon caliber and myelin sheath size [62–64], we next asked whether growth of axons in caliber might coincide with the observed remyelination and restoration of normal myelin sheaths, as previously suggested [35]. We first found that axon caliber does not change in treated or control animals during the period of demyelination, i.e. from 5-11dpt (mean perimeter of the largest 40 axons: at 5dpt, controls 3.32 μm ± 0.60 μm and treated 3.13 μm ± 0.62 μm; at 7dpt, controls 3.30μm ± 0.39μm and treated 3.19μm ± 0.46μm; at 11dpt, controls 3.21μm ± 0.23μm and treated 3.33μm ± 0.68μm; Fig 9). However, unexpectedly, we found that the average perimeter of the largest 40 reticulospinal axons grew in caliber by approximately 25% between 11 and 16 dpt in both control and treated animals, such that at 16dpt, the mean perimeter of the largest 40 axons grew to 4.34 ± 0.63μm in controls and 4.25 ± 0.59 μm in Mtz-treated animals. A two-way ANOVA including all the time points from 5dpt to 16dpt found a significant main effect of time point (p = 0.0003). The main effect of treatment condition and the interaction between time point and treatment were non-significant (p = 0.707) and p = 0.952, respectively; Fig 9). As indicated by the quantification in Fig 9B, the increase in axon caliber occurs specifically between the time points of 11dpt and 16dpt. To confirm that there is a significant difference between axon calibers between these time points, we compared the mean perimeters of the 40 largest axons at 11dpt and 16dpt. This showed that in controls the mean perimeter was 3.28μm ± 0.48μm and in treated animals 4.3μm ± 0.58μm (p = 0.0008, t test).

As we discuss below, this suggests the possibility that the active growth in axon caliber may promote the growth of myelin sheaths to their normal lengths and thicknesses.

## Discussion

The regeneration of myelin in MS and most animal models results in myelin sheaths shorter and thinner than normal, and the reasons for this remain unclear. We show essentially perfect regeneration of myelin following widespread demyelination of reticulospinal axons in the larval zebrafish spinal cord (see summary schematic in S3 Fig). Following treatment of our transgenic animals with the pro-drug metronidazole, we observe pathologies at the tissue and ultrastructural level that resemble those described in analogous mammalian models of oligodendrocyte cell ablation and demyelination [68–71]. Despite the ablation of 2/3rds of oligodendrocytes, gross disruption to tissue architecture, extensive demyelination, and a robust inflammatory response, we find that oligodendrocyte numbers are returned to precisely those of controls and that both tissue architecture and myelination are essentially indistinguishable just two weeks after the initial insult. Although this model of demyelination may not recapitulate the pathology or regenerative capacity of adult disease, the use of larval zebrafish allows extensive study of the cellular mechanisms involved in demyelination and remyelination.

### Does ongoing growth of axons in caliber drive regeneration of normal myelin sheaths?

The role of myelin in fine-tuning conduction and providing metabolic support to axons [75,76] suggests that regeneration of myelin sheaths of normal length and thickness may be required to restore these functions optimally. In fact, given the essential roles of myelin for axonal function and health, it remains quite unclear why default regeneration of myelin in the CNS should lead to the formation of short and thin myelin sheaths. Interestingly, previous studies indicate that this is not an immutable response to demyelination. For example, upregulation of MAPK signalling in oligodendrocytes has been shown capable of increasing myelin sheath thickness in both the healthy CNS and in response to demyelination [77], indicating that the regeneration of myelin sheaths of normal thickness in mammals is not an insurmountable task. Indeed, another recent study has brought in to question the conventional wisdom that default remyelination necessarily leads to the regeneration of diminutive myelin sheaths. Using a genetically encoded fluorescent reporter of myelin in the mammalian spinal cord, Powers et al., were able to follow remyelination along axons for extended periods of time following a spinal cord contusion injury. Importantly remyelination was monitored along axons that were spared by the initial injury but subsequently demyelinated, and not along those undergoing axonal de/regeneration, following injury. Remarkably, myelin sheaths of normal length and thickness were restored to demyelinated axons, albeit over a period of months [78]. This study challenged the premise that default remyelination necessarily results in short thin myelin sheaths, and suggests that regeneration of myelin may simply be a slow and protracted process. Indeed, this study highlighted the possibility that thin myelin observed in demyelination models could in principle represent ongoing demyelination and/ or ongoing remyelination, and not necessarily be a hallmark of a definitive endpoint representing remyelination. However, despite these observations, previous long-term studies of cuprizone-mediated demyelination analysed over the course of months have still found axons with abnormally thin myelin [11,79]. This begs the question as to whether the nature of demyelination may explain the difference in regenerative response. Indeed, the nature of remyelination following spinal cord injury induced demyelination may simply be distinct from primary disruption to myelin in animal models or chronic demyelination in complex disease such as MS. One critically important point to note is that in the spinal cord contusion model, axons grew significantly in caliber from one month to 3 months post injury, as myelin sheaths grew in length and thickness [78]. Despite the difference in model and time-scale, the observation of Powers et al., fits with our own, i.e. that regeneration of myelin sheaths of normal length and thickness occurs during ongoing growth of axons in caliber.

The hypothesis that growth of axons in caliber allows restoration of normal myelin sheaths requires further investigation. Regeneration of myelin sheaths of normal thickness has been observed in the optic nerve of young but not aged adult zebrafish following toxin-mediated demyelination [80]. It would be interesting to determine whether this restoration of myelin thickness correlates with active growth of axon caliber. Needless to say, experimental manipulations of caliber are required to experimentally test this hypothesis. Interestingly, a recent study showed that inactivation of Pten, a negative regulator of Akt signalling, in neurons could cell autonomously stimulate growth in axon caliber [62]. It would be interesting to test whether conditional inactivation of Pten in neurons could promote growth in caliber following induction of demyelination and whether this could stimulate the regeneration of myelin sheaths of normal length and thickness along such axons. In the PNS, the level of Neuregulin 1 on axons serve as a proxy readout of axonal caliber, and overexpression of Neuregulin 1 isoforms can indeed promote growth in thickness of myelin along individual axons [81]. In vivo, it is clear that individual oligodendrocytes can myelinate axons of very different caliber and locally generate myelin sheaths of the correct size [65,82]. Furthermore, introducing supernumerary large caliber axons can change the myelinating behaviour of individual oligodendrocytes [65]. However, we don’t know of any axonal signals in the CNS that strictly correlate with caliber and have such potential for regulating sheath growth, but this topic is under active investigation [62]. Very interestingly though, axon caliber in and of itself has recently been shown to represent a powerful regulator of myelination. Oligodendrocytes can generate myelin sheaths on inert synthetic axon-like fibers, but only do so on fibers above a certain threshold caliber of about 400nm [63]. Perhaps even more surprisingly, when oligodendrocytes are grown on fibers of mixed calibers the growth of individual myelin sheaths is tuned to the caliber of the fiber, i.e. longer myelin sheaths are formed on larger caliber fibers [64]. Together these data indicate that caliber in and of itself represents a signal that influences myelin sheath growth.

It is entirely possible that additional factors influence myelin sheath growth during remyelination. Axons may also grow in length in some cases during remyelination, although this response would likely be restricted to developing/ growing animals. Furthermore, cell intrinsic factors that regulate how oligodendrocytes behave at different stages of life [83] may also influence the myelin producing capacity of the cell. Extrinsic factors secreted from any number of cells in a tissue microenvironment may also regulate the growth capacity of the oligodendrocytes [84,85]. However, numerous recent studies have indicated that axonal signals can regulate myelin sheath length and thickness in the CNS [56,86–89], including during remyelination [90]. Therefore, we suggest that axonal growth and/ or associated signals represent the primary candidate regulators of regeneration of myelin sheaths of the normal size.

### Advantages and limitations of a larval zebrafish model of demyelination/ remyelination

We describe a model in which 2/3rds of oligodendrocytes in the larval zebrafish spinal cord can be ablated, leading to widespread myelin pathology and demyelination. We also observe an immune response, whereby macrophages/ microglia respond to the insult, move to the sites of myelin damage and appear to engulf myelin debris. Upon resolution of the immune response within just one week following cell ablation, oligodendrocyte and myelinated axon numbers are restored over the course of the following week, as are myelin sheathes of control dimensions. These events all occur in the context of a still developing animal [91], highlighting a remarkable capacity of the system to finely balance ongoing growth with a well-orchestrated bona fide regenerative response. Indeed the growth of axons in caliber during the third week of life occurs during general growth burst of the animal around this time [91].

One of the key advantages of studying demyelination and remyelination in a larval zebrafish is that demyelination of specific axons can be observed directly, due to the relative simplicity of the neuronal circuits myelinated early on in the developing zebrafish embryo. With the availability of new fluorescent reporters that allow assessment of myelin sheath number, distribution and length along single axons over time [56], one could in principle follow demyelination and remyelination of single axons *in vivo* over time. The distribution of myelin along axons is of critical importance in regulating conduction and function, and there is considerable diversity in the distribution of myelin along distinct axons, even to the extent that some myelinated axons have very large unmyelinated gaps between consecutive myelin sheaths (e.g. [92–96], which has implications relevant to the consequences of regenerating myelin appropriate in length and thickness to specific axons. Indeed it has also been proposed that the structure of nodes of Ranvier have important implications for myelinated axon function [44], and the precise nature of their reformation during remyelination is an area that warrants further study. The availability of additional reporters of axonal structure, organelles and other essential cargoes in zebrafish will allow one to assess the axonal response to demyelination with unprecedented phenotypic depth and resolution. As indicated by the consistent number and size of axons between control and treated animals, we did not observe any overt signs of axonal pathology following the acute cell ablation/ demyelination paradigm described in this study. However, the ability to carry out repeated metronidazole treatments over time should in principle lead to protracted demyelination, which may cause secondary axonal damage. Such longer-term imaging-based studies, coupled with ever-more sophisticated behavioural analyses in the zebrafish may also provide insight into how demyelination/ remyelination affects axonal health and circuit function at single cell resolution.

In the model described here we noted a very stereotyped response by the innate immune system, whereby microglia/ macrophages in the spinal cord increased in number and could also be seen to engulf fluorescent debris from myelinating cells. With the fluorescent reporter used in this study we could not distinguish the relative response of resident microglia and peripheral infiltrating macrophages to the sites of oligodendrocyte ablation/ demyelination. However, cell-type specific reporters and genetic regulators of these distinct macrophages and of other cells of the myeloid lineage would allow a more comprehensive assessment of innate immune system response to cell ablation and demyelination. The clearance of myelin debris is an important pre-requisite to myelin regeneration (e.g. [73,97,98]), but potential regulators are only beginning to be investigated [99]. Therefore, our model wherein macrophages can be visualised engulfing myelin debris could form the basis of discovery screens (genetic or chemical) to identify factors regulating myelin clearance.

This larval demyelination/ remyelination platform could also, in principle be used as a platform for discovery screens for regulators of myelin regeneration itself. However, it should be noted that the very fast regenerative response observed following resolution of the inflammatory response may impair our ability to identify positive regulators of remyelination, although identifying factors that impair remyelination may provide important insights. However, we should be clear that even with the early oligodendrocyte ablation carried out here (5–7 dpf), remyelination does not occur until between 2 and 3 weeks of larval life. During this period, it is difficult to standardise animal growth rates in the very large numbers required for discovery screens. Therefore, this platform may be better more suited to secondary screens and detailed studies of candidate regulators.

In summary, we describe a larval zebrafish model of demyelination and remyelination. This has allowed us to identify a possible explanation for how myelin sheaths of normal length and thickness could be regenerated during remyelination. Future studies using this and parallel systems will address the hypothesis that growth of axon caliber promotes the regeneration of myelin sheaths of normal dimensions, and help identify further mechanisms of remyelination *in vivo*.

## Supporting information

S1 Fig Metronidazole treatment does not affect oligodendrocyte number in and of itself. **A**. Lateral views of spinal cords of 7dpf Tg(mbp:nls-EGFP) larvae.
**B** Quantification shows the mean number of oligodendrocytes in a four-somite stretch of the spinal cord: in control: 83.33 ± 8.20 vs treated: 87.92 ± 13.64, p = 0.324. n = 12 for DMSO, 13 for Mtz.
(TIF)

S2 Fig Oligodendrocyte ablation results in extensive myelin vacuolation. **A**. Oligodendrocytes labelled with mbp:EGFP-CAAX in DMSO- or Mtz-treated Tg(mbp:mCherry-NTR) animals, before (top panel) or after (bottom panel) a two-day treatment with Mtz. Red arrowheads indicate putative myelin vacuoles.
**A**. Representative example of myelin vacuolation and disruption of spinal cord organisation, as visualised by the stable transgenic line Tg(mbp:EGFP-CAAX).
**B**. Brightfield images taken at 3dpt (top), where large vacuoles are clearly visible in the Mtz-treated animal (red arrowheads) and at 9dpt (bottom), when vacuoles are no longer detectible.
**C**. Representative electron micrographs of ventral hemi-spinal cords of DMSO- and Mtz-treated Tg(mbp:mCherry-NTR) animals. The control image shows the typical organisation of the spinal cord at this age, whereas the treated image contains numerous large fluid-filled vacuoles (labelled with red letters V) which disrupt the overall structure of the spinal cord.
Scale bars in A-B: 20μm.
(TIF)

S3 Fig Summary of events in Tg(mbp:mCherry-NTR) model. Timeline to illustrate the sequence of events following treatment of Tg(mbp:mCherry-NTR) larvae with metronidazole.
(TIF)
