# Supplementary figures and images for: Regeneration of myelin sheaths of normal length and thickness in the zebrafish CNS correlates with growth of axons in caliber
Source: PLoS One. 2017 May 25;12(5):e0178058. doi: 10.1371/journal.pone.0178058 (PMC5444792; doi:10.1371/journal.pone.0178058)

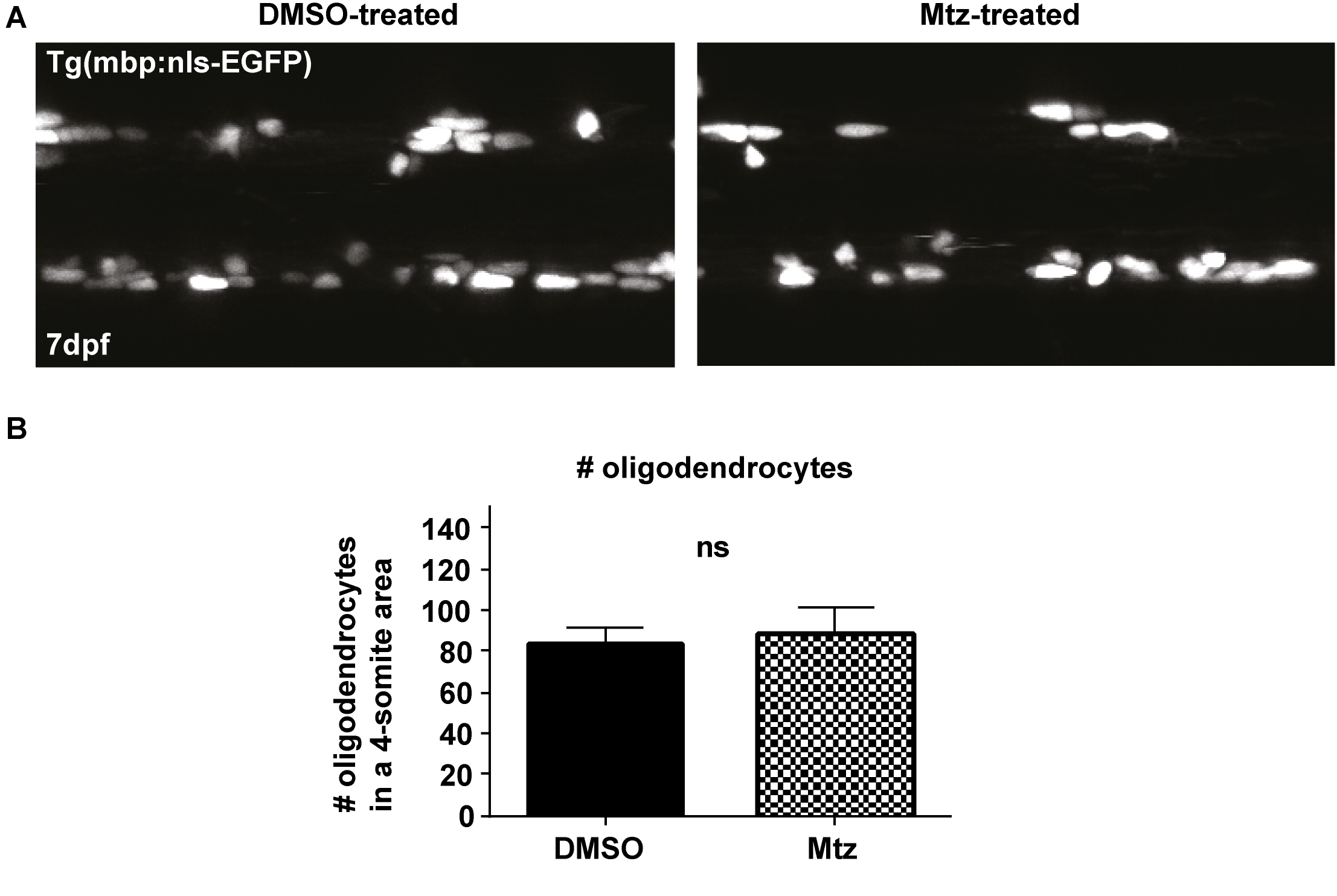

Supplement: S1 Fig — A. Lateral views of spinal cords of 7dpf Tg(mbp:nls-EGFP) larvae. B Quantification shows the mean number of oligodendrocytes in a four-somite stretch of the spinal cord: in control: 83.33 ± 8.20 vs treated: 87.92 ± 13.64, p = 0.324. n = 12 for DMSO, 13 for Mtz. (TIF) [file pone.0178058.s001.tif]

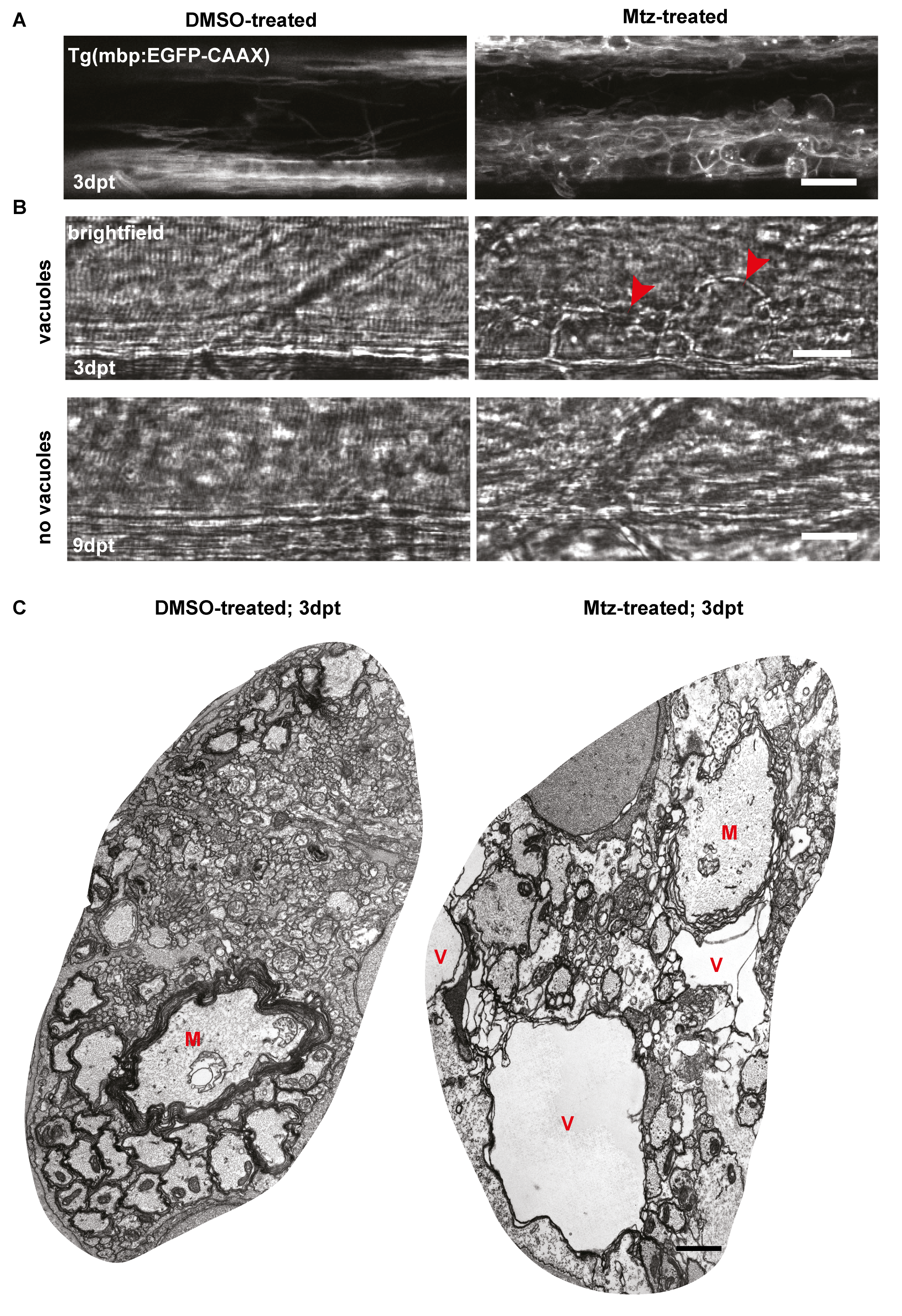

Supplement: S2 Fig — A. Oligodendrocytes labelled with mbp:EGFP-CAAX in DMSO- or Mtz-treated Tg(mbp:mCherry-NTR) animals, before (top panel) or after (bottom panel) a two-day treatment with Mtz. Red arrowheads indicate putative myelin vacuoles. A. Representative example of myelin vacuolation and disruption of spinal cord organisation, as visualised by the stable transgenic line Tg(mbp:EGFP-CAAX). B. Brightfield images taken at 3dpt (top), where large vacuoles are clearly visible in the Mtz-treated animal (red arrowheads) and at 9dpt (bottom), when vacuoles are no longer detectible. C. Representative electron micrographs of ventral hemi-spinal cords of DMSO- and Mtz-treated Tg(mbp:mCherry-NTR) animals. The control image shows the typical organisation of the spinal cord at this age, whereas the treated image contains numerous large fluid-filled vacuoles (labelled with red letters V) which disrupt the overall structure of the spinal cord. Scale bars in A-B: 20μm. (TIF) [file pone.0178058.s002.tif]

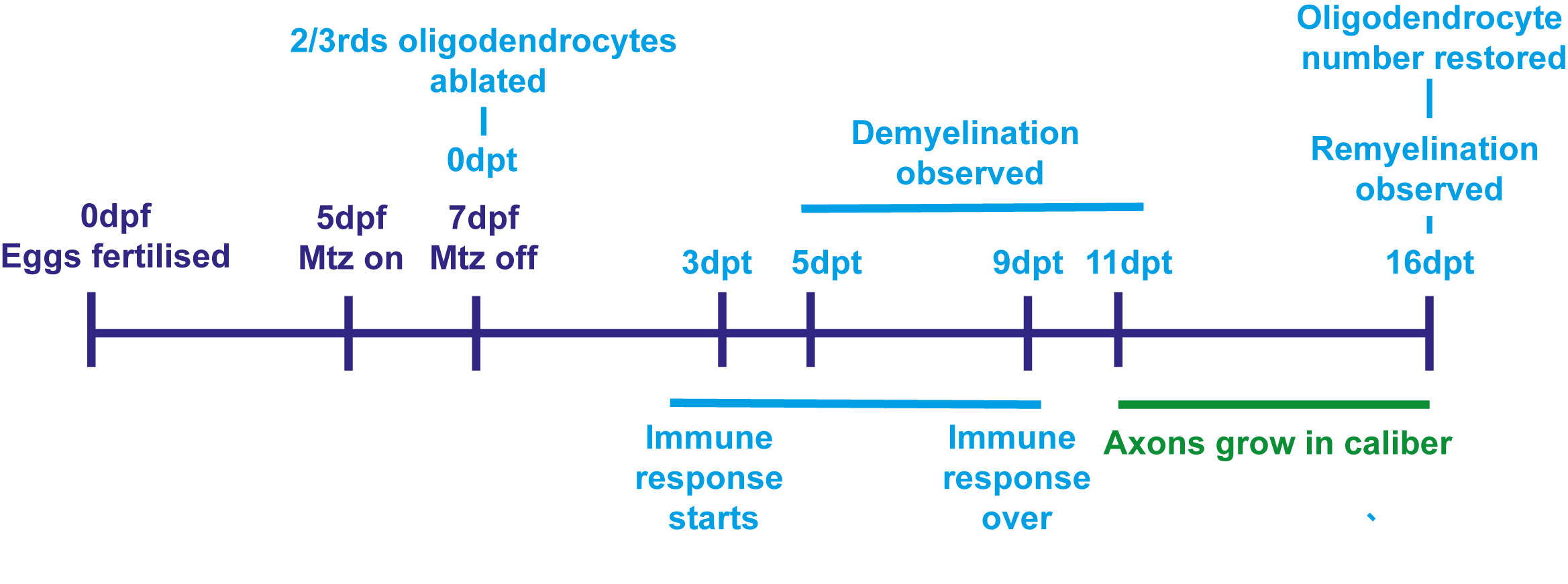

Supplement: S3 Fig — Timeline to illustrate the sequence of events following treatment of Tg(mbp:mCherry-NTR) larvae with metronidazole. (TIF) [file pone.0178058.s003.tif]
